# St Louis Dental Society

**Published:** 1889-02

**Authors:** Wm. Conrad

**Affiliations:** Corresponding Secretary; 321 N. Grand Ave.


### ST. LOUIS DENTAL SOCIETY.

The annual meeting of this society was held at the office of Dr. A. J. Prosser, 3109 Olive st., Wednesday evening, Jan. 2d, and the following officers were elected for 1889:

*President*, Dr. A. J. Prosser; *Vice-President*, Dr. J. Warren Wick; *Recording Secretary*, Dr. Jessie E. Grasheider; *Corresponding Secretary*, Dr. William Conrad; *Treasurer*, Dr. Henry Fisher; *Publication Committee*, Drs. H. H. Keith, John G. Harper and J. B. Vernon; *Committee on Ethics and Elections*, Drs. J. B. Newby, William N. Morrison and A. H. Fulley.

There were seventeen meetings held and fourteen papers presented during 1888.

WM. CONRAD,

321 N. Grand Ave. *Corresponding Secretary.*

---

As we go to press the news of the serious illness of Dr. F. Searle, of Springfield, Mass., comes to hand. His illness, though of short duration, is considered fatal.—ED.

